# Identifying Acute Cardiac Hazard in Early Drug Discovery Using a Calcium Transient High-Throughput Assay in Human-Induced Pluripotent Stem Cell-Derived Cardiomyocytes

**DOI:** 10.3389/fphys.2022.838435

**Published:** 2022-04-25

**Authors:** Hua Rong Lu, Mohamed Kreir, Van Ammel Karel, Fetene Tekle, Danny Geyskens, Ard Teisman, David J. Gallacher

**Affiliations:** ^1^ Global Safety Pharmacology, Predictive, Investigative and Translational Toxicology, Nonclinical Safety, Beerse, Belgium; ^2^ Discovery and Nonclinical Safety Statistics, Statistics and Decision Sciences, Quantitative Sciences, Janssen R&D, A Division of Janssen Pharmaceutica NV, Beerse, Belgium

**Keywords:** stem cells, cardiomyocytes (hiPS-CMs), cardiac hazard risk, HTS assay, translation

## Abstract

**Introduction:** Early identification of cardiac risk is essential for reducing late-stage attrition in drug development. We adapted the previously published cardiac hazard risk-scoring system using a calcium transient assay in human stem cell-derived CMs for the identification of cardiac risks recorded from the new hiPSC-CM line and investigated its predictivity and translational value based on the screening of a large number of reference and proprietary compounds.

**Methods:** Evaluation of 55 reference drugs provided the translation of various pharmacological effects into a single hazard label (no, low, high, or very high hazard) using a Ca^2+^-sensitive fluorescent dye assay recorded by -by FDSS/µCell Functional Drug Screening System (Hamamatsu on hiPSC-CM line (FCDI iCell Cardiomyocytes^2^).

**Results:** Application of the adapted hazard scoring system in the Ca^2+^ transient assay, using a second hiPS-CM line, provided comparable scoring results and predictivity of hazard, to the previously published scoring approach, with different pharmacological drug classes, as well as screening new chemical entities (NCE’s) using a single hazard label from four different scoring levels (no, low, high, or very high hazard). The scoring system results also showed minimal variability across three different lots of hiPSC-CMs, indicating good reproducibility of the cell line. The predictivity values (sensitivity and specificity) for drug-induced acute cardiac risk for QT-interval prolongation and Torsade de pointes (TdPs) were >95% and statistical modeling confirmed the prediction of proarrhythmic risk. The outcomes of the NCEs also showed consistency with findings in other well-established *in vitro* and *in vivo* cardiac risk assays.

**Conclusion:** Evaluation of a large list of reference compounds and internal NCEs has confirmed the applicability of the adaptations made to the previously published novel scoring system for the hiPSC-CMs. The validation also established the predictivity for drug-induced cardiac risks with good translation to other established preclinical *in vitro* and *in vivo* assays, confirming the application of this novel scoring system in different stem cell-CM lines for early cardiac hazard identification.

## Introduction

Early detection and elimination of new chemical entities (NCEs) with potential cardiac safety risks in drug discovery is essential for reducing late-stage attrition. Importantly, this strategy can reduce the potential risk for participants in clinical studies, and reduce wasted investment costs in late-stage development and increase the likelihood of advancing safe and effective novel therapeutics. The primary focus of cardiac safety within the current regulatory guidelines is to avoid drug-induced potentially life-threatening arrhythmias such as torsade de Pointes (TdP) (Gintant et al., 2016; Lu et al., 2017). TdP is associated with prolongation of QT-interval, which is primarily linked to the inhibition of human ether-a-go-go current (hERG), which encodes the pore forming a-subunit of rapidly activating delayed rectifier current potassium current (I_Kr_), in various species including human. In addition to QT prolongation, other pharmacological actions can result in drug-induced cardiac toxicities such as QT shortening (Lu et al., 2008) and QRS widening (Lu et al., 2010), which are also associated with conduction abnormalities and even companying with non-TdP-like ventricular tachycardia (VT) or ventricular fibrillation (VF). These later drug-induced cardiac toxicities, which are not related to QT prolongation (or with hERG inhibition), are also to be covered during early drug discovery in pharmaceutical research and development (R&D).

Human-induced pluripotent stem-cell-derived cardiomyocytes (hiPSC-CMs) are now applied as part of early safety de-risking of NCEs (Authier et al., 2017) (2005, Ovics et al., 2020) and were evaluated in one of the workstream elements of the Comprehensive *in vitro* Proarrhythmia Assay (CiPA) initiative (Colatsky et al., 2016; Gintant et al., 2016). Within the CIPA workstream, the Myocyte Team, coordinated by the Health and Environmental Sciences Institute (HESI) and the US Food and Drug Administration (FDA), conducted studies with 28 reference drugs known to have different potential risks in humans using various technologies including microelectrode array (MEA) and voltage-sensing optical action potential recording in hiPSC-CMs (Blinova et al., 2018). The International Conference on Harmonization (ICH) final Concept paper on S7B and E14 Q&A (2018) also supports the use of human stem cell assays in preclinical safety screening Food and Drug Administration, HHS (2005). Additionally, a Ca^2+^ dye t assay to define the drug-induced QT prolongation and proarrhythmic risks of CIPA drugs was also successfully validated in hiPSC-CMs across multiple test sites (Lu et al., 2019).

Uses of Ca^2+^-sensitive fluorescence dyes in Stem cell-CM cultures provide imaging of intracellular Ca^2+^ transients, resembling both the rise and decay of cytosolic Ca^2+^ during a cardiac action potential (Blanchette et al., 2018) (Bootman et al., 2018). The Ca^2+^ transient model in hiPSC-CMs gives multiple indirect electrophysiological readouts, and could be considered a high-throughput screening (HTS) assay for detecting drug-induced cardiac liabilities. Earlier research works showed the translational value of the Ca^2+^ transient measurement assay on hiPSC-CMs (CTCM) assay, based on the outcomes of reference drugs with known liabilities in humans (Lu et al., 2015; Rast et al., 2015; Bedut et al., 2016; Dempsey et al., 2016; Zeng et al., 2016; Watanabe et al., 2017; Kopljar et al., 2018b). The readout of Ca^2+^ transient assay is similar to that of the voltage-dye readout on hiPSC-CMs as a HTS screening assay (Mathur et al., 2015; Lu et al., 2017), but the utility of the calcium assay (CTCM) is less expensive (Kopljar et al., 2018b; Lu et al., 2019). Furthermore, hiPSC-CMs are known to have more relevant pharmacological responses in comparison with the existing hERG-mediated ion current assay and certain nonhuman action potential studies in isolated cardiac tissue or Langendorff isolated heart assays (Takasuna et al., 2017). The application of the CTCM assay, which permits HTS evaluation of a large number of NCEs, has been facilitated by the introduction of a score algorithm, which can be used to rank NCEs based on the degree of hazard in a pharmacological response manner on the assay ((Kopljar et al., 2018b). However, as the algorithm is cell-line specific, the key challenge is to implement the scoring system for other hiPSC-CM lines. A straightforward approach is needed to introduce the scoring system for different lines of hiPSC-CMs. In the current study we used data obtained from iCell Cardiomyocytes^2^ to develop the algorithm for this cell line using the same principles that were employed to an earlier cell line (Cor.4U- Cardiomyocytes) (Kopljar et al., 2018b). Ideally, the HTS CTCM data from another hiPSC-CM line should be adapted to provide a similar unified score that should be able to rank NCEs, based on hazard score in a concentration-dependent manner. Additionally, we have demonstrated the predictivity and sensitivity of the CTCM assay in this cell line for assessing drug-induced acute cardiac risks, and the statistical modeling predictor for long QT and TdP. We also investigated the translational value of the CTCM assay for prediction of liabilities in other established *in vitro* and *in vivo* nonclinical assays, e.g., hERG assay, isolated cardiac tissue (*in vitro*), and anesthetized guinea pigs (*in vivo*).

## Methods

**FIGURE 1:**
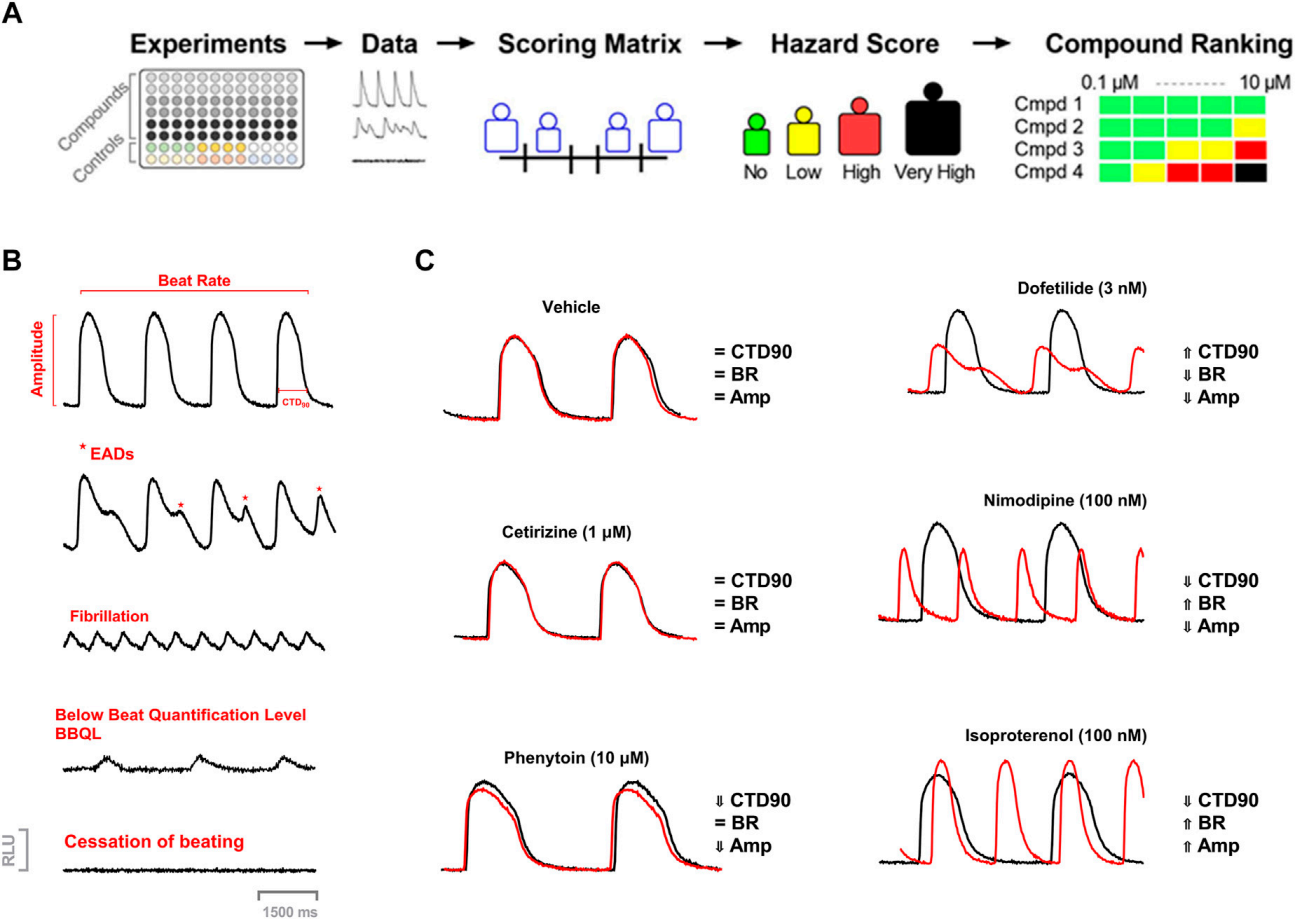
**(A)** Workflow of Acute Cardiac Hazard Identification. Drugs were tested in hiPSC-CMs using calcium dye imaging. Responses in hiPSC-CMs were analyzed based on several measured parameters to define the range of hazard scores. This strategy for determining concentration-dependent hazard and ranking of compound candidates is represented schematically. **(B)** Examples of calcium transient recordings showing effects on different measured parameters, arrhythmic responses, and abnormal function: calcium transient duration at 90% of repolarization (CTD_90_), beat rate (BR), amplitude (Amp); EAD*-like* arrhythmia, fibrillation*-like* arrhythmia, low amplitude transients (below beat quantification level; BBQL) and cessation of beating. **(C)** Example Ca^2+^ transient tracings showing tracings of vehicle, dofetilide (3 nM), nimodipine (100 nM), and isoproterenol (100 nM) at 30 min.

The concept of cardiac hazard identification used in the current study was similar to the approach used earlier with the Cor.4u cell line (cardiomyocytes) (Kopljar et al., 2018b). In the present study, we used another cell line (iCell^®^ Cardiomyocytes) to introduce the acute hazard score system to rank compounds based on the various parameters in the Ca^2+^ transient assay. (Figure 1).

### Cell Culture and Reagents for the Maintenance of hiPSC-CMs

A commercially available hiPSC-CM cell line was used (iCell Cardiomyocytes^2^: Catalog number R1017, Kit/lot 12,012; Fujifilm Cellular Dynamics Inc., Madison, WI, United States) for the study. Cells were pre-plated and seeded in fibronectin-coated 96-well plates at a density suited to form a monolayer and maintained in culture in a stage incubator (37°C, 5% CO_2_) according to the instructions of the cell provider. The experiments with test drugs were carried out 5–7 days after plating cells on the plate to have living, beating monolayer cardiomyocytes. The beating monolayer was taken from frozen vials iCell Cardiomyocytes^2^ (≈5 million cells/vial), which were plated onto three 96-well plates (≈50K/well).

To investigate whether there could be lot-to-lot variability with hiPSC-CMs, we tested 55 drugs, known cardiac risks, in one lot of iCell Cardiomyocytes^2^ (Lot 12,012), 19 common reference drugs in another lot of iCell Cardiomyocytes^2^ (Lot105091) as well as in iCell Cardiomyocytes (Lot 103,674) (non-square cells). The experiments with test drugs were carried out 5–7 days for iCell Cardiomyocytes^2^ and 12–15 days (for iCell Cardiomyocytes) after plating cells to have living, beating monolayer cardiomyocytes (≈5 million cells/vial/96-well plates) (≈50K/well). Cells from these lot numbers were from the same donor (Apparently healthy normal, female, Caucasian, age <18) (according to FUJIFILM Cellular Dynamics).

### Calcium Transient Measurements

The method for the Ca^2+^ transient assay used in the present study has been described previously (Zeng et al., 2016; Kopljar et al., 2018a; Kopljar et al., 2018b).

On the day of the experiment, the culture medium in the 96-well plates containing the monolayers of hiPSC-CMs was replaced with Tyrode’s solution (Sigma, No. T2397) supplemented with 10 mM HEPES together with KCl to yield isokalemic (4.2 mM K^+^) conditions.

The Ca^2+^-sensitive fluorescence dye Cal-520™ AM (Cat. no.21131; AAT Bioquest) was used to capture the intracellular Ca^2+^ transients in hiPSC-CMs. The protocol used was as described in Kopljar et al. (2018a). Briefly, Cal-520 was incubated for 70 min followed by a wash-out with supplemented Tyrode’s solution and a 30-min recovery before starting the experiments at 37°C. On the day of the experiment, these solutions were further diluted with the supplemented Tyrode’s solution. Compounds were added automatically using the Functional Drug Screen System (FDSS/µCell; Hamamatsu, Japan) head stage by adding 100 µl of the 2-fold concentrated compound solution to wells with hiPSC-CMs already containing a volume of 100 µl of the experimental solution, reaching the final intended test concentration in 0.1% DMSO.

The spontaneous beating activity of hiPSC-CMs was assessed through measurement of the Ca^2+^ fluorescence signal integrated over the whole well. Ten minutes before starting the recordings, the experimental plates were put into the FDSS system for stabilization and a baseline recording was captured for 4 min followed by compound addition. The effect of the compounds was recorded around 30 min after compound addition. The Ca^2+^ transient signals were sampled every 16 ms (frame rate of 62.5 Hz) in hiPSC-CMs maintained at 37°C during the acquisition time. Calcium transient duration at 90% of repolarization (CTD_90_) is considered to be a surrogate for action potential duration at 90% repolarization or QT-interval of the electrocardiogram (Spencer et al., 2014). In addition, beat rate (BR = peak count/min) and amplitude (amp = difference between max. and min of every beat) were measured, and incidence of cellular arrhythmias, e.g., EAD-like activity (considered a surrogate of TdP), cessation of beating (∼ stop beating), and below beating below quantification level (caused by low amplitude of the Ca^2+^ transient or by ectopic beat: BBQL) were also recorded (Figures 1B,C).

### Compound Selection, Drug Dilution, and Addition

The compounds and their respective test concentrations were selected based on the CiPA compound list (Blinova et al., 2018 #240) and from our earlier publication (Kopljar et al., 2018b). Four DMSO stocks for each drug concentration were prepared and either used on the same day or aliquoted and frozen. Concentrated (2-fold) testing solutions (50-fold for sequential dosing) for each concentration were prepared freshly on the day of testing by diluting DMSO stocks into the experimental medium. Two-fold dilution was done when drugs were added to the experimental well to achieve targeted concentration. For sequential dosing, DMSO concentrations were adjusted sequentially up to 0.1% at the highest concentration to achieve the targeted concentration of each drug. Each compound concentration was tested in 5-6 replicates.

Dofetilide (3 nM), isoprenaline (100 nM), and nimodipine (300 nM) were used as the positive controls, while cetirizine (300 nM) was used as the negative control. A total of 55 reference compounds were used in this study with different known clinical cardiac risks.

### Statistical Analysis for the Determination of the Score System

For all individual experiments, delta percent (Δ%) at 30 min with respect to the baseline value was calculated [e.g., ∆% for CTD_90_ = (CTD_90_ 30 min-CTD_90_ 0 min)/CTD_90_ 0 min) × 100]. The following two statistical approaches were used to make decisions about a compound’s effect.

First, the tolerance interval (TI)-based categorization, where the Δ% values of all (pooled) DMSO wells (experiments in this study) were centered around zero (corrected with the mean) and the parametric 90–95% (*p* = 0.90 & 1-α = 0.95) tolerance interval (TI) was calculated (Liao et al., 2005). TIs indicate an interval where, with a certain confidence level (1- α = 0.95), a specified proportion (*p* = 0.90) of a sampled population falls. The lower and upper limits based on the TI values were used as cut-offs for the vehicle-corrected net effect (ΔΔ%) values of the compounds to categorize them. These ΔΔ% net effects were the values obtained after normalization for baseline (Δ% vs. baseline) per well and the aggregated compound-treated wells are vehicle corrected by subtraction with the aggregated Δ% of the corresponding DMSO wells from the same plate [e.g., ΔΔ% = median Δ% drug - median Δ% DMSO]. The categorization was made first for each concentration and then aggregated per compound and test site. If a net effect (ΔΔ%) at a given concentration of a compound was below the lower limit of the TI, then it was categorized as “decreased”; if the net effect was within the DMSO TI then it was categorized as “no effect,” and if the net effect was above the upper limit of the TI then it was categorized as “increased.” At the compound level, a compound was categorized as “increased” if it was in a category “increased” in at least one of the four concentrations. Similarly, a compound was categorized as decreased if it was in a category “decreased” in at least one of the four concentrations. Based on TI values, we determined the cut-off values for the hazard score system (Kopljar et al., 2018b).

Data are expressed as means ± SEM. All statistical analyses were performed using SAS^®^ software (Copyright^©^ 2002–2012 SAS Institute Inc.).

### Sensitivity, Specificity, and Predictive Value of Potential Cardiac Risks in the Assay

Significant prolongation of CTD_90_ observed in this assay was considered a surrogate for drug-induced QT prolongation and TdP risk. Since some drugs cause QT prolongation in humans at overdoses and/or in combination with other drugs, the predictivity values were calculated at free maximal plasma concentration in human (fC_max_) and also at a threshold of 10 X fC_max_ or 30X fC_max_. The list of reference drugs that have QT-prolongation and potential risk for TdP in humans was taken from the ParmaPendium^®^ (www.pharmapendium.com) and from our recent publication (Kopljar et al., 2018b). A true positive (TP) was defined as QT prolonging drugs that significantly prolonged CTD_90_, while true negative (TN) was defined as non-QT prolonging drugs in humans that did not prolong CTD_90_ at a tested concentration. A false negative (FN) was defined as a known QT-prolonging drug that did not prolong CTD_90_, while a false positive (FP) was defined as a non-prolonging QT drug that significantly prolonged CTD_90_ at > 30-fold fC_max_. Sensitivity was calculated as the % of QT-prolonging drugs correctly predicted based on CTD_90_ prolongation in this assay [TP/(TP + FN)]. Specificity was calculated as the % of non CTD_90_ prolonging drugs correctly predicted as non-QT prolonging drugs [TN/[(TN + FP)]. Positive predictive value (PPV) was calculated as: PPV = [TP/(TP + FP)], while negative predictive value (NPV) was NPV = [TN/(TN + FN)].

### Statistical Modeling of Drug Proarrhythmic Potential Based on its hiPSC-CMs Effects

The modeling used in this study was primarily based on the seven predictors from the measurement parameters in MEA as given in the CiPA publication (Blinova et al., 2018), with a slight modification. Data on effects of 55 drugs with known clinical risk of TdP obtained from all experimental sites were used to construct a model that would predict TdP risk category of a drug based on its effects on hiPSC-CMs. Seven modified endpoints from hiPSC-CM experiments used as potential model predictors (Blinova et al., 2018) were: Predictor 1, drug-induced cessation of beating and early afterdepolarization (EAD) at any concentration; Predictor 2, describes the ability of a drug to induce arrhythmia*-like* events (EAD) in over 40% of wells in hiPSC-CMs; Predictor 3, CTD_90_ (ms) at the first drug concentration with statistically significant (*p* ≤ 0.05) prolongation or shortening; Predictor 4, reflects the amount of drug-induced repolarization prolongation (CTD_90_% of baseline) at the lowest concentration where statistically significant change from the baseline was observed or maximum prolongation at any of the studied concentrations; Predictors 5 and 6, account for concentrations of a drug relative to its fC_max_ when prolongation of CTD_90_ or incidence of arrhythmia-*like* event was first observed; and Predictor 7, an estimated amount of CTD_90_ prolongation that a drug would induce at its clinical fC_max_. We added predictor 8 that includes drug-induced cessation of beating in ≥40% of wells at any concentration, which added an extra value to detect drug-induced slow conduction time or inhibiting spontaneous beating.

Logistic regression models (Model 1 and Model 2) were then used (high or intermediate risk vs. low risk–Model 1, and high vs. low and intermediate-risk vs. low risk–Model 2) on all eight risk predictors according to the CiPA publication (Blinova et al., 2018).

### Lot-to-Lot Variability

To investigate lot-to-lot variability in hiPSC-CMs, we tested 19 drugs with known clinical cardiac risk in other lots of iCell Cardiomyocytes^2^. Fifty-five reference drugs were tested in iCell Cardiomyocytes^2^ -Lot12012, 18 reference drugs were tested in iCell Cardiomyocytes^2^ (Lot105091), and 18 reference drugs were tested in iCell Cardiomyocytes (Lot103674) (nonsquare cells).

### Comparison of Outcomes Between the Current CTCM Human Assay in hiPSC-CMs (iCell-Cardiomyocytes^2^) and the Earlier Stem Cell-Derived Cardiomyocytes (Cor.4u^®^ Cardiomyocytes)

To investigate the reproducibility of the cardiac hazard risk predictions from the different cell lines, we compared the present data with the results of 55 reference drugs obtained from hiPSC-CMs (iCell Cardiomyocytes^2^) to the data obtained from another cell line which was published previously (Kopljar et al., 2018b).

### Evaluation of the Translatability of the Scored Hazard Potential of NCEs in the Calcium Transient Assay in hiPSC-CMs to Other Nonclinical *in Vitro* and *in Vivo* Cardiac Safety Assays Including Animal Models

We also evaluated the translational predictability of the risk scores of NCEs in the CTCM assay to our complementary cardiac safety models: hERG assay, isolated rabbit wedge preparation (*ex vivo*) (Lu et al., 2016), and anesthetized guinea-pig (*in vivo*) models (Kopljar et al., 2018b) using the Software - SPEcII (Unlimit-IT, Belgium). All the experiments involving the use of animals have been conducted in accordance with “The provision of the European Convention” on the protection of vertebrate animals that are used for experimental and other scientific purposes, https://rm.coe.int/168007a67b.

## Results

### Acute Cardiac Hazard Scoring System

We applied our previously reported strategy and methodology for the development of a cardiac hazard scoring system based on phenotypic readouts from Ca^2+^ transient imaging to a different hiPSC-CM cell line (iCell Cardiomyocytes^2^) (Figure 1A). For a qualitative evaluation of hiPSC-CMs, each test plate (i.e., 96 unique experiments) contained 0.15 DMSO controls, together with several control drugs (cetirizine, isoprenaline, nimodipine, and dofetilide). These drugs have different pharmacological classes and were served as a pharmacological reference set for the development of the hazard scoring system. Cetirizine is a real negative control known not to have any clinical cardiac liabilities. Nimodipine was used as a calcium channel antagonist and isoprenaline is a beta-adrenergic agonist. Dofetilide is a hERG blocker associated with QT prolongation and proarrhythmic TdP risk in humans. Pharmacological effects in hiPSC-CMs were investigated after a 30-min incubation period and normalized against the respective baseline recording, yielding a Δ% change in CTD_90_, BR, and Amp.

**FIGURE 2:**
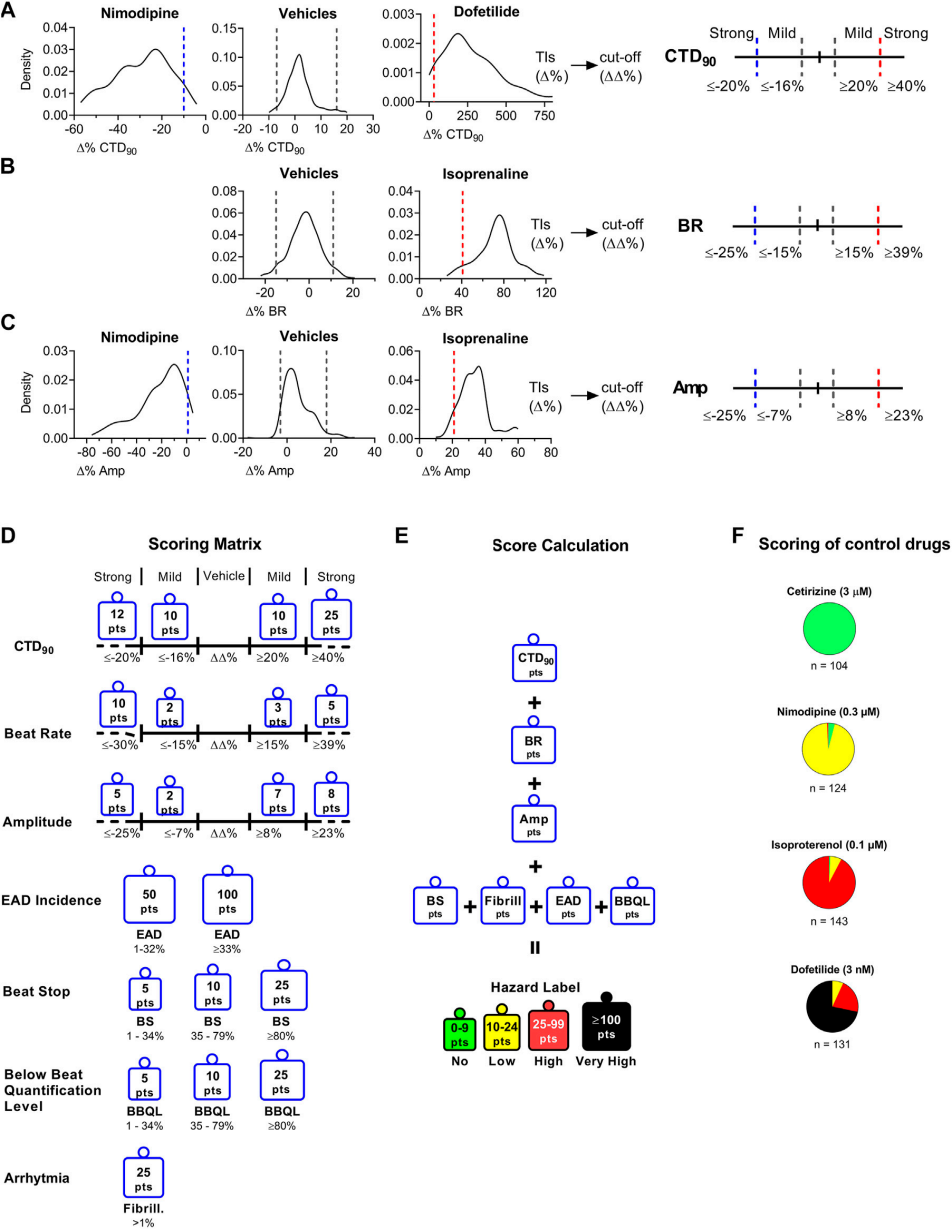
Determination of Cutoff Values Using Statistical Tolerance Intervals (TIs) to Build a Hazard Scoring Matrix. The TIs (dashed lines within graphs) for vehicles and positive controls to determine bidirectional cutoffs for CTD_90_ to be mild or strong effects (Panel A), BR (Panel B), and Amp (Panel C). TIs from vehicle (n = 368) were applied to define the “no effect” cutoffs as well as the cut-off between “no effect” and “mild effect.” Panel **(A)**: Nimodipine (300 nM; n = 173) and dofetilide (3 nM; n = 178) were used to determine “strong” CTD_90_ shortening and prolongation cut-offs, respectively. Panel **(B)**: Isoprenaline (100 nM; n = 171), and 0.1% DMSO (n = 368). TIs were used to define “strong” BR increase. Panel **(C)**: Nimodipine and isoprenaline were used to define “strong” amplitude decrease and increase, respectively. Tolerance intervals (Δ %) were corrected for vehicle offset to determine the cut-offs (ΔΔ%). **(D)**: Scoring points for hazard score identification: The scoring matrix shows a points card where for each parameter a weighted score is given depending on the size and direction of the ΔΔ% effect. **(E)**: Calculation of hazard level based on the sum of points across all parameters. **(F)**: Pie charts showing the scoring of various reference drugs over multiple studies. Cetirizine (3 μM; n = 104), isoproterenol (100 nM; n = 143), nimodipine (300 nM; n = 124), dofetilide (3 nM; n = 131). *n* indicates the number of studies; each study contained 4–8 individual experiments.

The current hazard scoring outcome was largely similar to our previously reported scoring system for the Cor.4u cell line (Kopljar, 2018 #245, based on the TIs and its modified cut-off values of key parameters: CTD_90_, beat rate, amplitude, and incidence of arrhythmic events (EAD and cessation of beating). The scoring matrix was determined based on the cut-offs between the different effect zones for each parameter (Figure 2). The “no effect” zone shows minimal changes in a parameter that is within vehicle variability, whereas “mild” and “strong” zones are determined from mild to large changes bidirectionally (e.g., CTD_90_ shortening and prolongation). The cut-off values are net changes (ΔΔ% changes vs. baseline and vehicle), which are based on the statistical tolerance intervals (TIs) (Δ% changes vs. baseline). TIs indicate an interval where, with a certain confidence level, a specified proportion of a sampled population falls. Vehicle treatments showed low variability in Δ% change for CTD_90_, BR, and Amp with respect to baseline (Figure 2). Cardiac hazard scoring for the tested compound at a given concentration was given into the following risk classification hazard score (color labels): “No” (green), “Low” (yellow), “High” (red), and “Very High” (black). No hazard labeling means that a tested compound’s effects were within the vehicle variability. Low hazard score indicates that the effects were outside the vehicle variability, with = low or limited risk. High hazard score gives a strong concern whereas the very high hazard score suggests potentials to cause cardiac arrhythmias-EADs.

### Investigation of the Hazard Scoring System

Based on the outcomes of the positive/negative controls and the reference drug list used in another cell line (Cor.4U) (Kopljar, 2018 #245), we validated the results in this new hiPSC-CM cell line (iCell Cardiomyocytes^2^) which was essential for using the scoring system for hazard identification of NCEs. Figure 2F shows the hazard distribution for control drugs (at a single concentration) tested in numerous studies. Cetirizine at 1 µM (16-fold the fC_max_) as a negative control was exclusively identified to be a no hazard agent. Nimodipine at 300 nM (15-fold fC_max_) (n = 124) and isoproterenol (n = 143) at 100 nM (63-fold fC_max_) are both cardio-active agents that were identified within the low and high hazard zones (95 and 92%, respectively). Dofetilide at 3 nM (2-fold f C_max_) (n = 131) was correctly identified as yellow (8%), high (red color) (21%), or very high (black color) (71%) hazard score.

**FIGURE 3:**
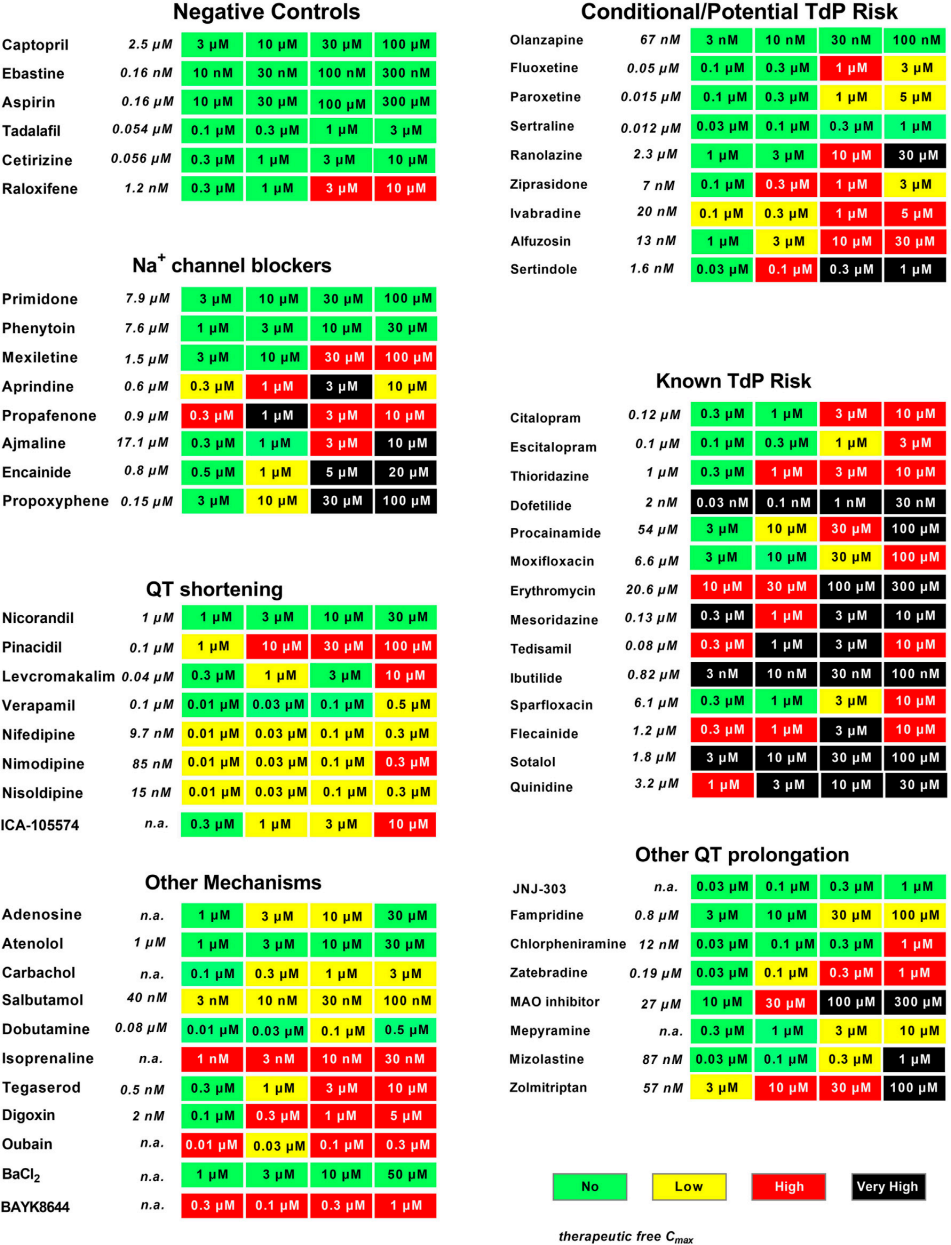
Evaluation of reference drugs for cardiac hazard. Fifty-five reference drugs were scored for cardiac hazard using the scoring matrix. “Known TdP”, “conditional TdP”/potential TdP′ groups show the marketed medicine containing these warnings in their FDA label, as well as some reference drugs that are known to block Na^+^ channels, shorten QT, or have other mechanisms acting on cardiac receptors or other ion channels. Concentrations were tested according to the therapeutic fC_max_ (shown in italics), as described in our previous publication (Kopljar et al., 2018b). n. a, not available.

**FIGURE 4:**
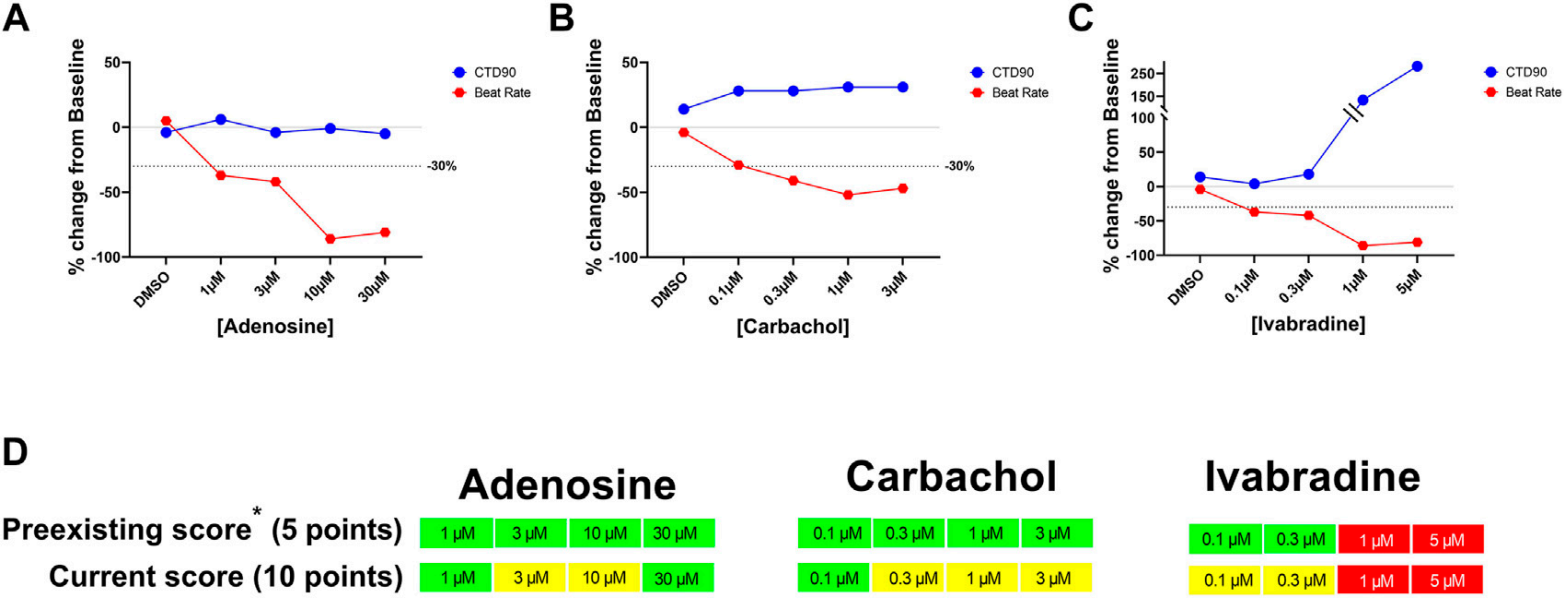
Concentration-dependent effects of adenosine, carbachol, and ivabradine on beat rate and CTD90 in hiPSC-CMs. Panels **(A–C)**: Concentration-dependent changes in CTD_90_ and beat rate. Panel **(D)**: Identification of significant decrease in the beat rate in the current hazard scoring system by adding extra score points for decrease in beat rate (i.e., ≥30%-ΔΔ%).

Following up, we validated the hazard scoring (as defined in Figure 3) using 55 reference drugs and their respective known clinical risk relative to their fC_max_. These reference drugs are categorized based on their pharmacological mechanism or level of cardiac side effects in men (Figure 4). Indeed, negative control drugs have no reports of cardiac liabilities in humans and are expected to be proven to be “no hazard” for concentrations up to 10- to 30-fold fC_max_. All negative controls were found to have “no hazard” (green). The only special case was raloxifene at clinically irrelevant concentrations (3–10 μM; ≥2500-fold the clinical fC_max_).

We investigated the potential hazard scoring system for drugs, known to be associated with QT prolongation and a certain degree of TdP risk in humans (Figure 3) (These drugs were categorized into two groups according to the FDA label or described in www.torsades.org: conditional TdP/potential TdP risk, and known TdP incidence. In cases of overdose, drug–drug interactions, or in certain high-risk individuals (Woosley et al., 2018). The rank order of incidence of EADs or degree of prolongation of CTD_90_ could be found to be co-related well to known TdP drugs > potential TdP risk or “Conditional TdP risk.” Only the antidepressant olanzapine was not found to be with any hazard level, most likely because of the fact that the highest tested concentration was 100 nM, which is around fC_max_. This concentration might be too low, since olanzapine was also only found to significantly inhibit hERG at a concentration of 6 µM, which is at 90-fold fC_max_ (Titier et al., 2005).

Although the main focus within preclinical safety pharmacology in most pharmaceutical companies is on drug-induced QT prolongation and TdP, other pharmacological mechanisms affecting different cardiac ionic currents can also result in cardiac toxicity such as, e.g., QRS widening or QT shortening. Na^+^ channel blockers such as primidone and phenytoin, which are considered relatively safe in humans, had little impact on hiPSC-CMs (Green score) and another Na^+^ channel blocker, mexiletine, caused some cessation of beating only at a very high concentration (100 µM = 67x fC_max_), resulting in a Red score in the current study, similar to that in the CIPA validation study (Blinova et al., 2018). However, most Na^+^ blockers also inhibit hERG current at similar concentrations, which are readily detected as CTD_90_ prolongation and incidence of EADs leading to “low” to “very high” risk (Figure 3).

Ca^2+^ channel antagonists resulted in strong responses in hiPSC-CMs, showing a marked decrease in amplitude companying with CTD_90_ shortening and pronounced beat rate increase. The cardiac I_KATP_ channel opener and the hERG activator (ICA-105574), which shortened CTD_90_, were also characterized in the present study.

Additional pharmacological mechanisms including beta-adrenergic agonists (isoproterenol, dobutamine, and salbutamol), Na^+^/K^+^ ATPase inhibitors (digoxin and ouabain), calcium channel activator (BAYK8644), and a nonselective 5-HT_4_ agonist (tegaserod), which were withdrawn from the market due to adverse cardiovascular events, were also validated in the present study and showed similar acute hazard scoring outcome as determined in Cor.4U cell line. As expected, BaCl_2_ (an I_K1_ inhibitor) had no effect on hiPSC-CMs since it is known that most current commercial hiPSC-CMs lack functional I_K1_ channels (immature phenotype) (Goversen et al., 2018; Pourrier and Fedida 2020). Similarly, I_Ks_ inhibitor (JNJ-303) was not detected in hiPSC-CMs (Kopljar, 2018 #245).

In the previous score system with Cor.4u cell line (Kopljar et al., 2018b), significant drug-induced decrease in beat rate did not significantly impact hazard score because the maximal decrease in beat rate was only adding 5 points while the threshold for the minimal hazard score (Yellow; low risk) was 10 points. In the current updated scoring system, significant decreases induced by the muscarinic acetylcholine receptor M_2_ agonist (carbachol), adenosine receptor agonist (adenosine), and funny channel current (I_f_) inhibitor (ivabradine) were identified as potential “low” risk (Yellow) by adding 10 points for the decrease in beat rate by ≥ −30% (delta/delta from baseline and 0.1% DMSO control) (Figure 4). With this update in the scoring paradigm for changes in beat rate, we identified 29 out of ∼3,000 screened reference and proprietary compounds that decreased beat rate by ≥ 30%. These 29 compounds include carbachol, adenosine, zatebradine, ivabradine, arecoline, methacholine, ibutilide, flecainide, and other internal NCEs.

### Lot-to-Lot Variability

To investigate the lot-to-lot variability in responses/scores of hiPSC-CMs provided by the vendor, some of the reference drugs that prolong QT and/or have TdP risks (total of 19 compounds) were tested in different lots.

The mean baseline Ca^2+^ transient parameter values were amplitude (3,470, 2,854, and 2017 RLU), beat rate (28, 26, and 34 beats/min), and CTD_90_ (947, 1,006, and 790 ms) for lot 12,012 (iCell Cardiomyocytes^2^) (n = 2,463), lot 105091 (iCell^2^ Cardiomyocytes^2^) (n = 521), and lot 103674 iCell Cardiomyocytes) (n = 400), respectively. There were no incidences of EADs or cessation of beating at baseline. The responses to the positive controls (isoproterenol, nimodipine, and dofetilide) and the negative controls (cetirizine or DMSO) were qualitatively similar among the three lots. For example, dofetilide (3 nM) prolonged CTD_90_ by 305% in lot 12,012 (n = 144), 330% in lot 10,591 (n = 28), and 234% in lot105091 (n = 20), and incidence of EADs was similar among the two iCell Cardiomyocytes^2^ lots and the iCell Cardiomyocytes: 63, 71, and 65% incidence of EADs, respectively.

**FIGURE 5:**
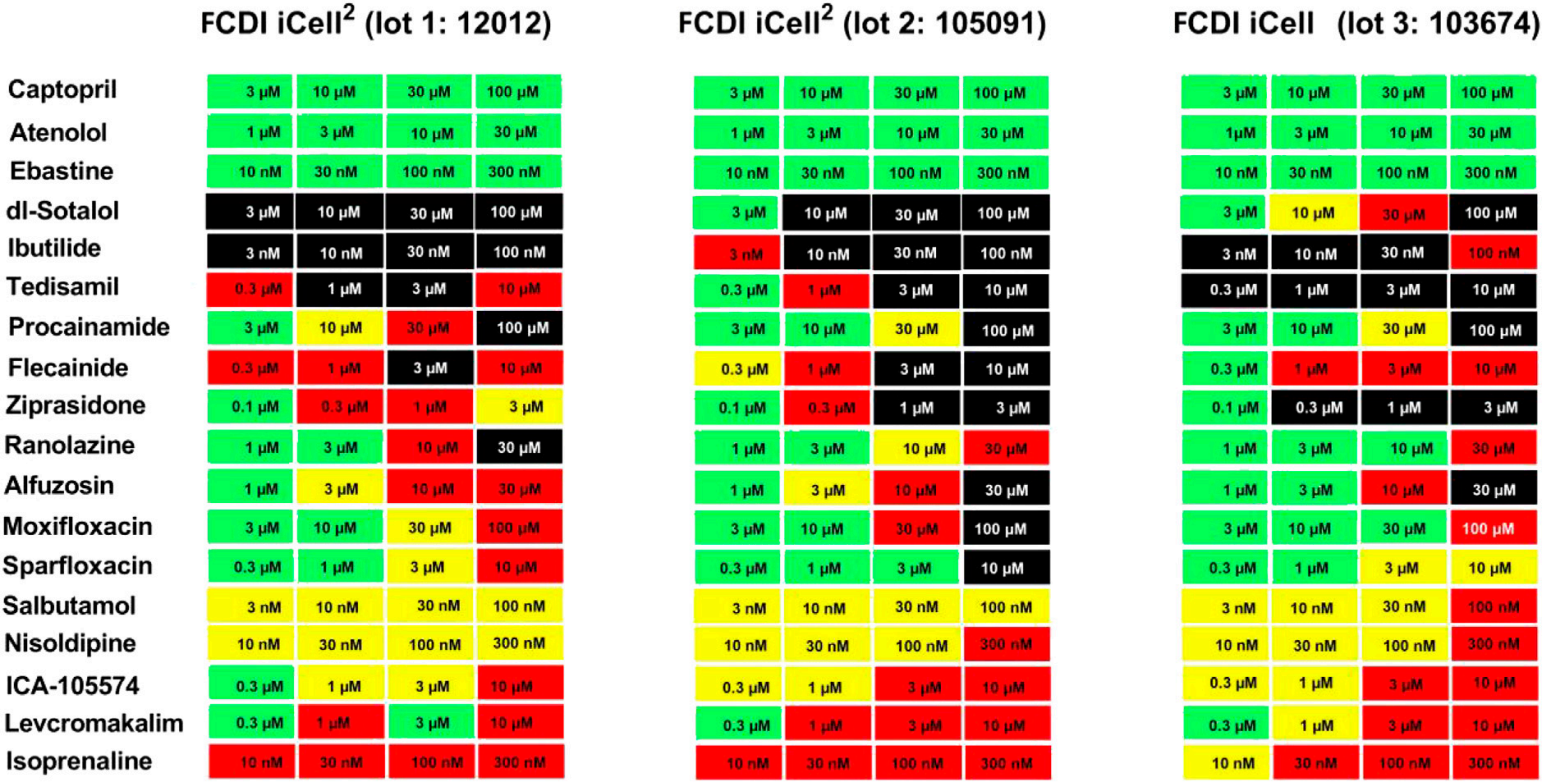
Evaluation of Cardiac Hazard Risk of Nineteen Reference Drugs in Two iCell-Cardiomyocytes^2^ Lots (12012 and 105091) and one iCell Cardiomyocyte Lot (103674). Reference drugs were scored for cardiac hazard using the described scoring matrix. “no TdP risk,” “TdP risk,” and “other mechanism” groups represent drug categories based on FDA labels for marketed drugs.

Responses to three drugs that do not prolong QT or have no TdP risk (captopril, ebastine, atenolol) were similar among the iCell Cardiomyocytes^2^ and iCell Cardiomyocytes^2^ lots: “no hazard”/green color, Figure 4). Interestingly, the cardiac risk scores for 10 drugs known to prolong QT and induced TdP were also similar in two lots of iCell Cardiomyocytes^2^ and iCell Cardiomyocytes. However, EAD incidence for dl-sotalol was much higher in iCell Cardiomyocytes (10 µM) compared to the two lots of iCell-Cardiomyocytes^2^ (3 and 10 µM). Five reference drugs with other mechanisms of acute cardiac risk (hERG activator, IK_ATP_ channel opener, Ca^2+^ antagonist, and Na^+^ channel blockers) were also similar among the three lots with slight variations acceptable for any *in vitro* assay (Figure 5).

### Analysis of Sensitivity, Specificity, and Predictivity Values

We analyzed the potential acute cardiac risks of 55 reference drugs with known degrees of risk in humans using the current hazard scoring system based on changes in beat rate, amplitude, CTD_90_, and incidence of arrhythmias. We compared the cardiac risk of these 55 reference drugs based on our scoring system to the potential for clinical QT-prolongation/TdP risk, QT-shortening, QRS-prolongation, or all potential cardiac arrhythmias (i.e., non- TdP like VT/VF, bradycardia, etc.). Based on the numbers of true positives (TP),true negatives (TN), false positives (FP), and false negatives (FN), we calculated sensitivity (TP/(TP + FN)), specificity (TN/(TN + FP)), and balanced accuracy (TP + TN).

**FIGURE 6:**
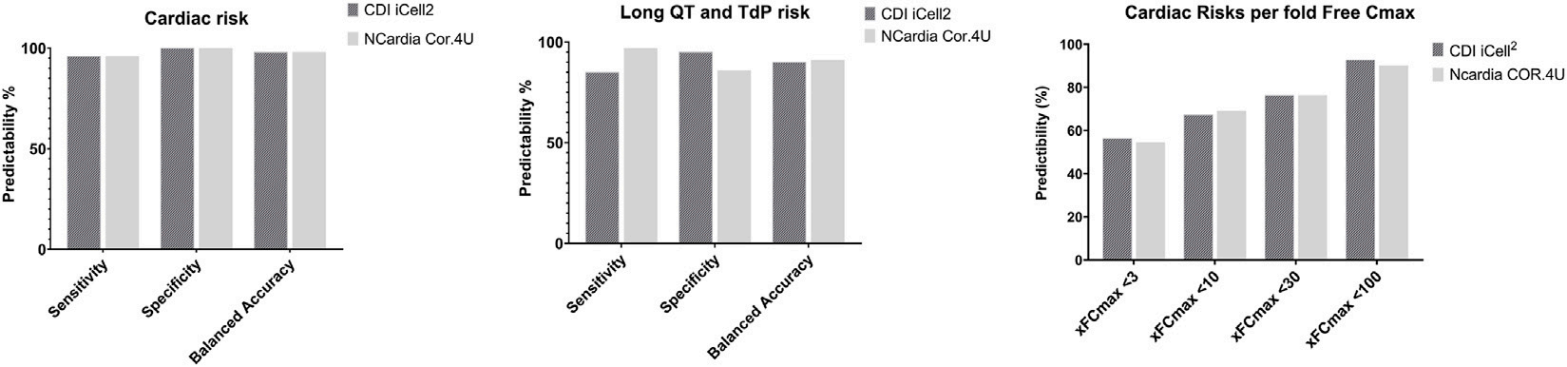
Comparison of Sensitivity, Specificity, and Balanced Accuracy of Cardiac Risk Assessment Between iCell Cardiomyocytes^2^ and Cor.4U cell line. Left Panel: Overall cardiac risk; Middle Panel: QT-prolongation and potential TdP risk; Right Panel: Cardiac risk predictability based on different multiples of fC_max_ level of the 55 reference drugs known to have potential cardiac risks in humans.

In summary, the total cardiac risks prediction was at 95%: 10 no cardiac risk drugs were all negative (green score), and for QT-prolongation/TdP risk on this CTCM assay with a balanced predictivity of 90%, sensitivity of 85%, and 95% specificity (Figure 6). FP was at 0 out of 43 while FN was at 2 out of 12 compounds (BaCl2 and JNJ-303: experimental drugs).

### Comparison of the Acute Cardiac Hazard Cardiac Risks Between the Current Cell Line-hiPSC-CMs (iCell Cardiomyocytes^2^) and Cor.4U Cell Line in the Ca^2+^ Transient Assay

**FIGURE 7:**
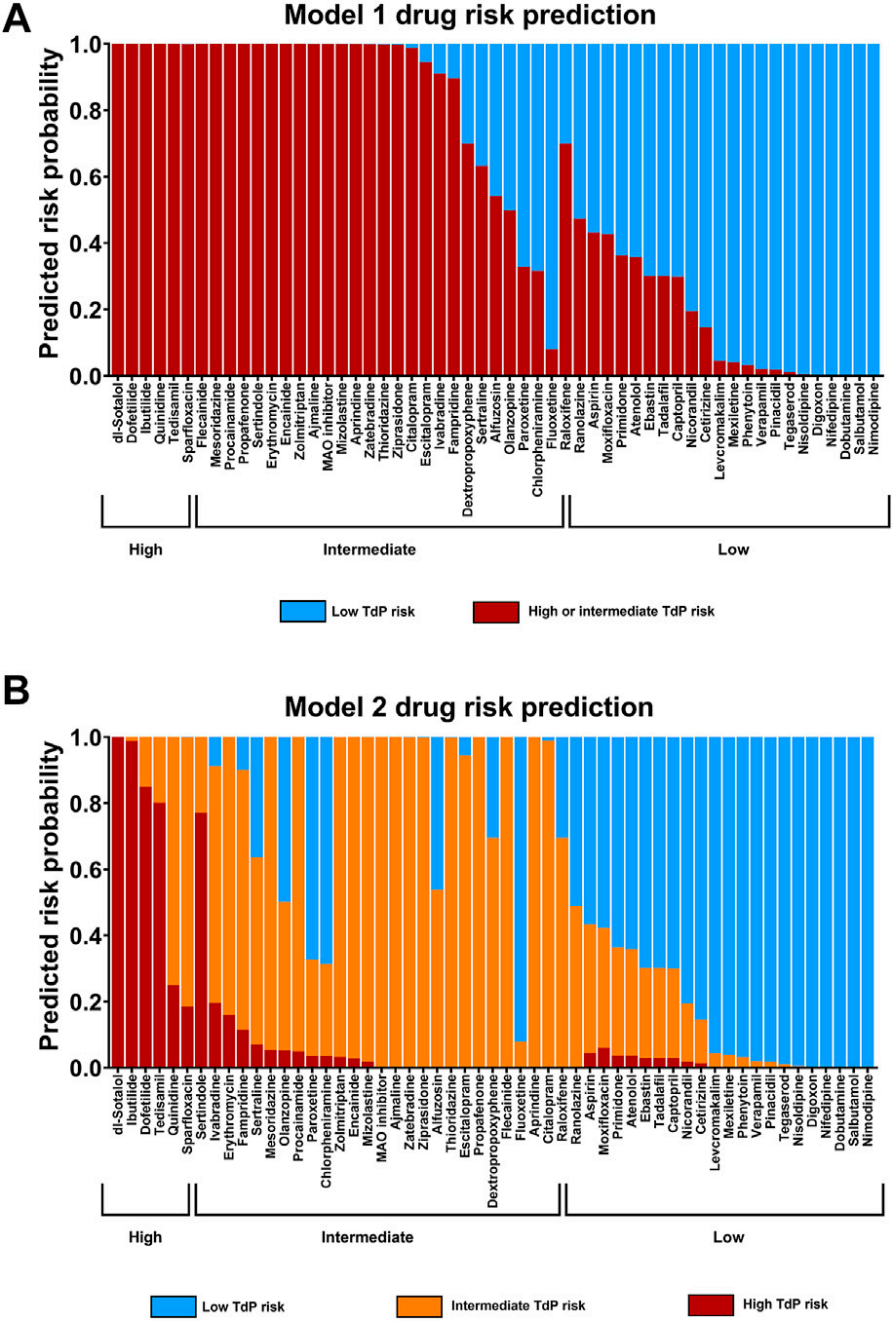
Statistical Modeling of Cardiac Risk. **(A)** Model 1 (dichotomous model): prediction of TdP risk category as either low or intermediate and high risk combined (averaged). **(B)** Model 2; ordinal model prediction as low, intermediate, or high TdP risk category. Both models are based on the predictors published in the CiPA paper (Blinova et al., 2018), with a slight modification based on the nature of the current study.

Fifty-five reference drugs were tested in iCell Cardiomyocytes^2^ in the current study and compared to Cor.4U in the CTCM assay (Kopljar et al., 2018b). The acute cardiac hazard scores of these 55 reference drugs were similar between the 2 cell lines. The prediction values of acute cardiac risk and QT prolongation/TdP risk in both cell lines were >90% (Figure 5A). When taking into account the fold over its fC_max_ (Figure 7B): <10 -fold fC_max_: the predictive value with the Cor.4u cell line was slightly higher, and at <30 or <100 -fold fC_max_, the iCell Cardiomyocytes^2^ had slightly higher predictive values compared with the Cor.4u cell line. However, both cell lines were equal in defining cardiac risks in general.

### Statistical Modeling of Drug Proarrhythmic Potential

In addition, we used statistical modeling of proarrhythmic potential for the 55 drugs with known clinical risk for QT-prolongation and TdP based on the results from CTCM assay and hazard scoring system. Eight endpoints from the CTCM assay were used for potential mode predictors that include seven predictors defined in the CiPA publication ((Blinova et al., 2018)), and an additional predictor: cessation of beating was included (Lu et al., 2019): Predictors 1 and 2 indicate a test drug that elicited an arrhythmia-like event (i.e., early afterdepolarization: EAD): Predictor 1 (binary): did any drug induce EAD at the tested concentration [0 = no EAD; 1 = EAD)]; Predictor 2 (binary): EAD observed at any concentrations in ≥40% wells (0 = no; 1 = yes); Predictor 3: first drug concentration with statistically significant (*p ≤* 0.05) prolongation or shortening of repolarization; Predictor 4: defined the maximal drug-induced changes in repolarization (CTD_90_) observed at any concentration; Predictor 5: defined drug concentration (multiple of fC_max_) at the first statistically significant (*p* < 0.05) prolongation of CTD_90_; Predictor 6: accounted for the first drug concentration relative to its clinical fC_max_ to induce EAD; Predictor 7: had drug-induced changes in CTD_90_ (ms) at ∼ its free C_max_; Predictor 8 (binary): caused cessation of beating at any concentration in ≥40% of wells (0 = no; 1 = yes).

As described in the CiPA publication, two logistic regression models for the potential TdP risk group were used: Model 1: had high or intermediate risk *vs.* low risk and Model 2: had high risk *vs.* low risk and intermediate risk *vs.* low risk.

The results of the 55 reference drugs including a large set of CiPA compounds in the statistical model are shown in Figure 7. The models predicted well for high- and intermediate-risk drugs as well as for low-risk drugs with identified incidence in humans. There were very limited drugs overlapping between the intermediate- and low-risk drug (e.g., raloxifene). However, the concentrations used in the study were substantially higher compared with its f C_max_ in humans (1.2 nM). Raloxifene is a selective estrogen receptor modulator that is used for women with postmenopausal osteoporosis and is not associated with QT-prolongation in clinic. Raloxifene was reported to inhibit I_Kr_ with an IC_50_ at 1.1 µM and I_Ks_ with an IC_50_ at 4.8µM, and voltage-gated Na^+^ current with an IC_50_ at2.8 µM (Liu et al., 2010). This may explain the identified activity in the current assay at much higher concentrations compared with free C_max_ (>1,000 fold its C_max_) that could be associated with some risk for QT-prolongation.

### Assessment of the Hazard Potential of NCEs

**FIGURE 8:**
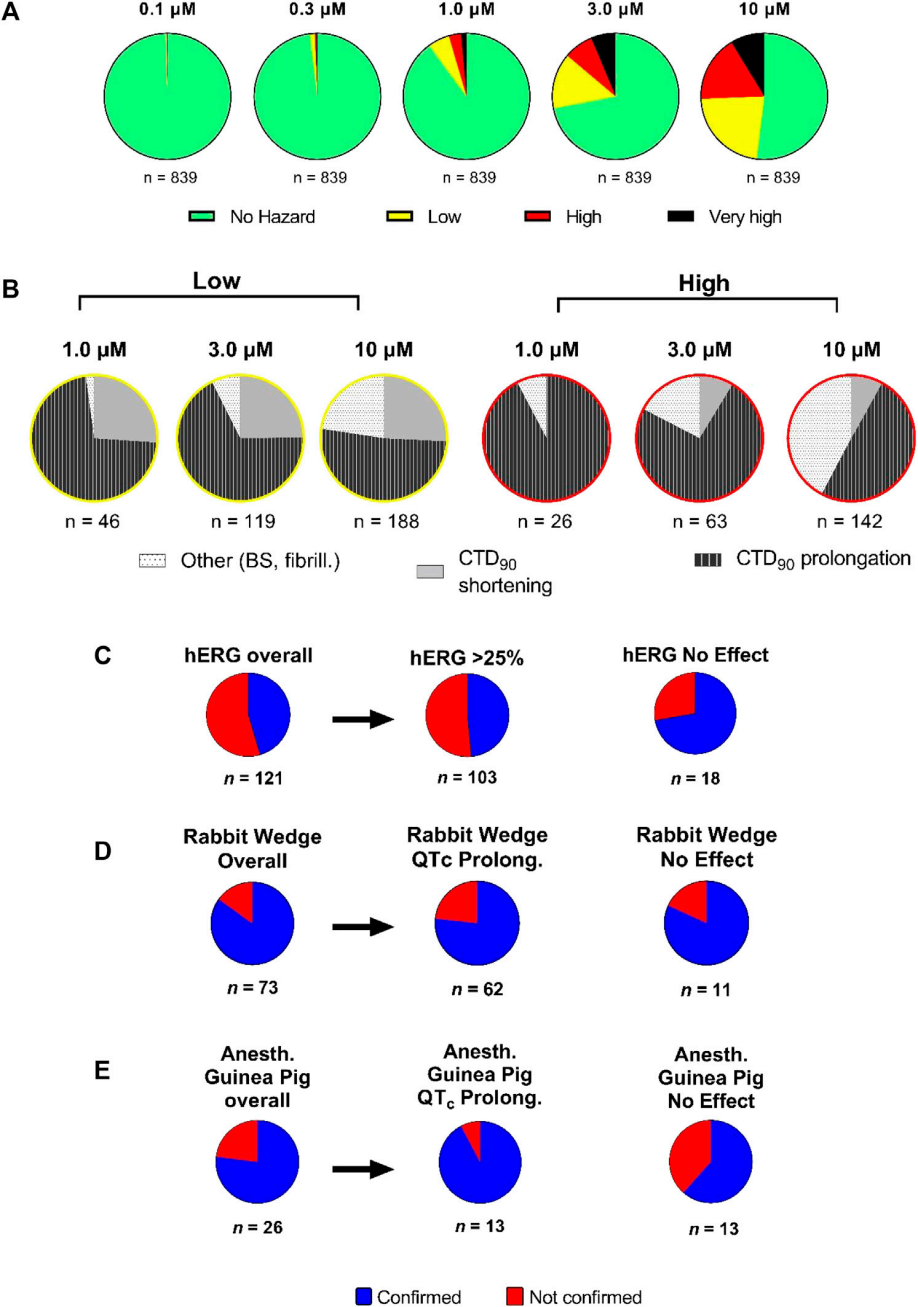
Hazard score investigation using the scoring system on Janssen NCEs in early R&D. **(A)** Pie charts showing the concentration-dependent distribution of different hazard score levels (*n* = 839). **(B)** Detailed analysis of proportion of effects on CTD_90_ (increase or decrease) and incidence of beat stop (BS) or fibrillation in low and “high risk” categories by concentration (1, 3, and 10 µM) on CTCM assay. Panels **(C–E)**: The outcome of acute cardiac hazard scores in CTCM assay was compared to the inhibition among 121 compounds in the hERG assay (Panel C), to the effects on QT interval or QRS duration among 73 compounds in isolated rabbit ventricular wedge assay (Panel D), and to the effects on QTcB interval and QRS duration among 26 compounds in anesthetized guinea-pigs (Panel E). In Panel D, the left side pie: labeled Overall: including effects on both QT interval and QRS duration on a total of 73 compounds. In Panel E, the left side pie: labeled Overall: including effects on both QTcB interval and QRS duration on a total of 26 compounds. Confirmed: matching outcome between the two assays. Not Confirmed: not matching outcome between two assays.

Sequentially, we investigated 839 NCEs including Janssen discovery research projects based on molecules (Figure 8A). Compounds were tested at a wide concentration range (0.1–10 µM). Hazard evaluation identified that the most of NCEs were classified within the “no hazard” group at concentrations less than 10 μM, with a portion showing risk at the highest concentration of 10 µM. 839 NCEs (54%) were found to be no hazard over the entire tested concentration range. Additionally, for compounds with identified hazard, the levels increased in a concentration-dependent manner. At 1 µM and higher, over 50% of the compounds did show some effects associated with a certain hazard level in hiPSC-CMs, with the “high hazard” mainly at 10 µM. The proportion in the “very high” compounds, linked to arrhythmic-like EADs, was relatively small at 10 µM with some incidence also observed at lower concentrations.

Although the hazard score classification is used as an initial indicator for potential for cardiac toxicities, further analysis in other models is necessary to help to better understand the pharmacological mechanisms involved, ultimately leading to a comprehensive integrated hazard identification. Therefore, we separated the low from high hazard groups (at 1, 3, and 10 µM) in terms of the directional effect on CTD_90_ (Figure 8B). CTD_90_ prolongation was mainly cause of hazard labeling among compounds for all hazard-concentration combinations (Figure 8B). At 1 and 3 μM, high hazard identification was mostly caused by CTD_90_ prolongation, and by the combination of CTD_90_ prolongation and beat stop (cessation of beating) at 10 µM.

### Translational Value of CTCM Human Assay Compared to hERG and QT Assessment

A sum of 121 compounds were tested in both the hERG patch-clamp and CTCM assays at the similar concentration ranges (Figure 8C). There were 66 compounds with no risk in the CTCM assay: 53 out of 66 compounds showed hERG inhibition ≥25% while 13 of 66 compounds showed no effect in the hERG assay. There were 55 of 121 compounds that had low to very high hazard score in the CTCM assay, and only 5 of these 55 compounds (9%) showed no effect in the hERG assay, while 50 of 55 compounds (91%) were confirmed active in the hERG assay, indicating that the drug-induced hazard scores were primarily due to inhibition of hERG current. The difference between the CTCM assay and the HERG assay could be caused by the effects on the CTCM assay not only mediated by affecting HERG current but also on other cardiac ion channels/receptors, or due to small % of false positive and negative on HERG assay.

Seventy-three compounds (acute hazard scores from the CTCM assay) were also tested in the isolated rabbit ventricular wedge preparation (Figure 8D). From 52 of these 73 compounds that had hazard scores in the CTCM assay, 96% (50 out of 52 compounds) had effects on either QT interval or QRS or both QRS and QT-interval in the isolated rabbit wedge assay, while only two (4%) had no effects on QRS or QT-interval in the isolated wedge preparation. Of the 21 of 73 compounds that had no hazard score in the CTCM assay 43% (9 of 21 compounds) were confirmed to have no effects in the isolated rabbit wedge preparation while 57% (12 of 21 compounds) had effects on QT interval and/or QRS: 8 compounds had QT effects; 3 had both QT and QRS effects, and 3 widened only QRS duration. However, the effects of these compounds on QT interval and QRS duration in the wedge preparation were relatively small.

Twenty-six compounds that had hazard scores in the CTCM assay were also tested in anesthetized guinea pigs (*in vivo*) (Figure 8E): Of the compounds that had no risk (green) in the CTCM assay, eight were also found to have no effects in anesthetized guinea-pigs in terms of changes in QRS duration and QTcB interval. One compound (terfenadine) slightly shortened CTD_90_ and caused cessation of beating in the CTCM assay, but significantly prolonged QRS duration and QTcB interval in the anesthetized guinea pig at much higher plasma levels than that of the CTCM assay. Terfenadine is known to have solubility issues *in vitro* and possibly resulting in less than nominal concentrations in the CTCM assay (Lu et al., 2012). In addition, terfenadine is rapidly metabolized in the liver to an active metabolite. Of seventeen compounds that scored yellow/red/black risk hazard scores in the CTCM assay, 71% were confirmed as positive in the anesthetized guinea pig. However, 29% of these 17 compounds (5 of 17 compounds) did not have significant effects in anesthetized guinea pigs in terms of changes in QRS duration and QTcB interval. Explanations for some of these discrepancies include: One compound slightly shortened CTD_90_ and tended to shorten QTcB interval *in vivo*; the free plasma levels of another four compounds (high plasma protein binding *in vivo*) were much lower than in the CTCM assay.

## Discussion

Human stem cell-derived CMs are now applied to evaluate cardiac liabilities as part of early de-risking in drug research and development (R&D). In our earlier publication ((Kopljar et al., 2018b)), we introduced a hazard scoring system to aid in the selection of new chemical entities (NCEs) devoid of acute cardiac risks. In the present study, we successfully applied the same principles of the previous hazard score system to a different hiPSC-CM line (iCell Cardiomyocytes^2^), using statistical TI (s) and cut-off values for the key parameters from Ca^2+^ transient fluorescence recordings, based on DMSO controls and a validation set of 55 clinically known reference drugs that included positive and negative controls for various parameters. Statistical analysis (tolerance intervals) on a large historical data set of vehicles and control drugs helped the development of a detailed scoring system with differentiation of size and direction of effect for each parameter. We documented and confirmed the utility of various parameters [(CTD_90_, beat rate, amplitude, cessation of beating, BBQL, fibrillation-like event and EADs) ], measured in spontaneously beating hiPSC-CMs to generate a hazard identification score from data of the different pharmacological classes of drugs. To our knowledge, this is a more extensive approach relative to most other studies using hiPSC-CMs, where the focus is exclusively on APD-related parameters (e.g., field potential duration or CTD_90_) associated with changes in the QT interval. The hazard coring system, based on the measurement of a Ca^2+^ transient in hiPSC-CMs (CTCM), can help detect and define cardiac hazards beyond drug-induced QT prolongation, such as QT shortening, beating rate changes, and slowing of conduction. This hazard score system can also identify potential hazards of drug-induced significant decreases in beating rate mediated by mechanisms such as muscarinic acetylcholine receptor M_2_ stimulation (carbachol), adenosine receptor agonist (adenosine), and I_f_ inhibitor (ivabradine), which was lacking in our previous hazard scoring system derived from Cor.4U-cell line Figure 8). With the newly updated hazard score system, by adding an extra 5 points to the risk score for drug-induced decrease in beat rate (total 10 points for decreases in rate by ≥ 30%), we now are able to identify this additional hazard as a low hazard score (Yellow) in 2.9% of 3,019 NCEs tested in this cell line. We have used the hazard scoring system to simplify the analysis of complex drug-induced effects on hiPSC-CMs and provide a scoring system to identify and select safe NCEs facilitating the progress of these molecules in early R&D. Interestingly, a decrease in beat rate ≥30% has limited propensity to prolong CTD_90_ (Figure 4): The prolongation of CTD_90_ is still within the cut-off values of DMSO controls in these cases, which indicates that the rate dependence of CTD_90_ in this *in vitro* assay is not the same as *in vivo*, where QT interval must generally be corrected for heart rate.

The discovery and development of novel NCEs is a long and expensive process, and there is a considerable rate of attrition resulting in part to a safety concern. Therefore, an *in vitro* assay capable of detecting safety issue early in discovery and development, particularly cardiac safety, is crucial for optimized candidate selection and reduction of human risk in clinical trials and with drugs approved for use in patients This assay and the scoring system defined and described in this study can be readily applied to select the best drug candidates based on safety.

Our validation set of 55 reference drugs included the large set of CiPA compounds, used in the FDA/HESI (Blinova et al., 2018) and JiCSA (Kanda et al., 2018) studies, and showed comparable risk assessment. The models were highly predictive for high- and intermediate-risk drugs as well as for low-risk drugs, based on known risks in humans. There were a few apparent examples of inconsistency between risk scores in the CTCM and level of risk in humans (e.g., raloxifene). Raloxifene is a selective estrogen receptor modulator that is broadly used in the treatment of postmenopausal osteoporosis and known to not prolong QT interval at therapeutic doses. In the present study, the concentrations of raloxifene used were higher than of its fC_max_ in humans (1.2 nM). Raloxifine presented with a red hazard score (high risk) but this was only observed at >2000 folds of its fC_max_. At ∼700–800 folds fC_max._ raloxifene had a green score (low risk), consistent with its clinical safety profile. Raloxifene has been reported to inhibit cardiac delayed rectifier K^+^ currents at high exposures (I_Kr_: IC_50_ = 1.1 µM and I_Ks_ = 4.8 µM), which may explain the high hazard score and potential for QT prolongation at >2000 folds its fC_max_.

Importantly, we also documented that there was very little lot-to-lot differences in hazard scoring results for 19 common reference drugs in iCell Cardiomyocytes^2^ (two lots) and iCell Cardiomyocytes (one lot).

Additionally, the specificity, sensitivity, and predictivity values for the cardiac risk assessment of 55 reference drugs were very high (>95%). The functional output of the current platform using Ca^2+^ transients enabled phenotypically relevant readouts of acute cardiac risk *in vitro*. The hazard scoring system predicted potential for long QT/TdP risk of 55 reference drugs as well as total cardiac risks (drug-induced short QT and VF*-like*) with over 95% specificity, sensitivity, and total balanced cardiac risk when compared with the known risk in humans. Importantly, the hazard scoring system predicted the cardiac risks of the 55 reference drugs mostly within the range of therapeutically relevant free plasma concentrations (fC_max_) (Figure 6). The set of 55 reference drugs in the study covered a broad range of different drug classes with different pharmacological mechanisms that include both cardiovascular and noncardiovascular drugs. Using such a large set of reference drugs we were able to establish a hazard scoring system that can be applied in the selection of safe NCEs early in the drug discovery process. Although a threshold of >10-(>30- or ≤100- fold) fC_max_ was used for analyzing the scoring and assessing the predictivity of the assay, it should be noted that in many *in vitro* assays concentrations up 30-fold fC_max_ are normally considered relevant for predicting cardiac effects in humans (Redfern et al., 2003). In both the present and the CiPA stem cell validation study (Lu et al., 2019), four concentrations were used to capture fC_max_ levels with 10- and 30-fold fC_max_ margins. Additionally, cardiac and noncardiac drugs including disopyramide, terfenadine, diphenhydramine, domperidone, fluoxetine, and sertindole, etc., are known to have functional cardiac effects in *vitro* assays at much higher concentrations than their fC_max_ level (>30–100 folds fC_max_) (Redfern et al., 2003). (Pacher et al., 2000; Eckardt et al., 2002).

To add value and gain confidence to the predictions and compound selection, screening results in this novel assay and scoring system for cardiac safety also need to be consistent with existing established early nonclinical screening assays, as well as more complex follow-up preclinical assays. Therefore, we evaluated the translational value of the current assay compared with other *in vitro* assays (such as the hERG current assay and the *ex vivo*-isolated rabbit left ventricular wedge preparation, as well as the anesthetized guinea-pig model, an established sensitive *in vivo* model to detect electrocardiographic risk (Testai et al., 2004; Lu et al., 2016)). The scoring system validation in the CTCM assay, based on reference and internal proprietary compound evaluation, was largely consistent with hERG assay results in terms of CTD_90_ prolongation, despite some false positives and negatives, as might be expected for compounds with more complex electrophysiological actions. The scoring outcome from the CTCM assay was also largely confirmed in anesthetized guinea pigs and the isolated rabbit wedge ventricular preparation (Figures 8C–E).

The scoring system that we developed provides a reliable way to readily identify the concentration-dependent level of cardiac risk by concentration for NCEs based on Ca^2+^ transient measurement in hiPSC-CMs using multiple functional parameters including beat rate, CTD_90_, and amplitude. However, despite the great promise of the current scoring system for predicting acute cardiac risks, a few limitations still exist. Similar to other *in vitro* assays, the current HTS assay does not detect potential effects of active drug metabolites, which would only be detected in an *in vivo* study or by directly testing the known active metabolite in the current assay. Poor solubility may also be an issue. Moreover, similar to any *in vitro* assay using hiPSC-CMs, these cells are not fully matured, although they express key cardiac genes and have comparable function of most cardiac ion channels and receptors, and this might influence the responses to some drugs in hiPSC-CM line. That could cause difference between hiPSC-CMs and isolated cardiac tissue assays.

Our current model validation data with 55 reference drugs showed predictions of drug-induced cardiac hazard score in hiPSC-CMs (iCell-Cardiomyocytes^2^) that were found to be similar to those in another cell line (Cor.4U Cardiomyocytes) (Kopljar et al., 2018b), indicating the adaptability and consistency of our scoring system approach. Screening of drug-induced effects in hiPSC-CMs can also be performed using different detection technologies, e.g., measurements of extracellular filed action potentials (Ando et al., 2017), impedance/MEA (Zhang et al., 2016; Bot et al., 2018), video motion imaging (Hayakawa et al., 2014), and voltage-sensitive dyes (Bedut et al., 2016). The principles used for the development of our scoring system could also be adapted for use in these other human cardiomyocyte systems using the same principles defined within this study.

In conclusion, the acute cardiac hazard score system applied to the Ca^2+^ transient assay using different hiPSC-CM cell lines should be useful for the identification and selection of NCEs for further investigation. The CTCM HTS assay system in our hands is user-friendly, and relatively inexpensive.

## Data Availability

The original contributions presented in the study are included in the article/Supplementary Material, further inquiries can be directed to the corresponding author.

## Abbreviations

Amp, amplitude; bpm, beats per minute; BR, beat rate; BS, beating stop (cessation of beating); BBQL, below beat qualitative level; CiPA, Comprehensive in vitro Proarrhythmia Assay; CTD, Calcium Transient Duration; DMSO, dimethylsulfoxide; EAD, Early Afterdepolarization; FDA, Food and Drug Administration; FDSS, Functional Drug Screening System; hERG, human ether-à-go-go-related gene; HESI, Health and Environmental Sciences Institute; hSC-CMs, human stem cell-derived cardiomyocytes; hiPSC-CMs, human induced pluripotent stem sell-derived cardiomyocytes; IKr, rapidly activating delayed rectifier potassium current; NCEs, new chemical entities.; R&D, Research & Development; RLU, Relative light unit; TdP, Torsades de Pointes; VF, Ventricular fibrillation.
